# Concentrated exosomes from menstrual blood-derived stromal cells improves ovarian activity in a rat model of premature ovarian insufficiency

**DOI:** 10.1186/s13287-021-02255-3

**Published:** 2021-03-12

**Authors:** Siwen Zhang, Boxian Huang, Peng Su, Qiyuan Chang, Pingping Li, Aixin Song, Xinyang Zhao, Zhengwei Yuan, Jichun Tan

**Affiliations:** 1 Center of Reproductive Medicine, Department of Obstetrics and Gynecology, Shengjing Hospital of China Medical University, No. 39 Huaxiang Road, Shenyang, 110022 Liaoning China; 2 Key Laboratory of Reproductive Dysfunction Diseases and Fertility Remodeling of Liaoning Province, No.39 Huaxiang Road, Shenyang, 110022 Liaoning Province China; 3 Department of Obstetrics and Gynecology, Affiliated Suzhou Hospital of Nanjing Medical University, Suzhou Municipal Hospital, Suzhou, 215002 China; 4 Center of Reproduction and Genetics, Affiliated Suzhou Hospital of Nanjing Medical University, Suzhou Municipal Hospital, Suzhou, 215002 China; 5 State Key Laboratory of Reproductive Medicine, Nanjing Medical University, Nanjing, 210029 China; 6 Key Laboratory of Research and Application of Animal Models for Environmental and Metabolic Disease, Shenyang, 117004 Liaoning Province China; 7 Key Laboratory of Health Ministry for Congenital Malformation, Shengjing Hospital of China Medical University, Shenyang, 117004 China

**Keywords:** Premature ovarian insufficiency, Menstrual blood-derived stromal cells, MenSCs, Exosomes, Rat model

## Abstract

**Background:**

Premature ovarian insufficiency (POI) is one of the major causes of infertility. We previously demonstrated that transplantation of menstrual blood-derived stromal cells (MenSCs) effectively improved ovarian function in a murine model of POI. Recent studies indicated that mesenchymal stem cell-derived exosomes were important components in tissue repair. In this study, we investigated the therapeutic effects of MenSCs-derived exosomes (MenSCs-Exos) in a rat model of POI and its mechanism in restoring ovulation.

**Methods:**

Ovaries of 4.5-day-old Sprague Dawley rats (SD rats) were cultured in vitro to evaluate the effects of MenSCs-Exos exposure on early follicle development. Furthermore, POI in rats was induced by intraperitoneal administration of 4-vinylcyclohexene diepoxide (VCD). Forty-eight POI rats were randomly assigned to four groups, each receiving a different treatment: PBS, MenSCs, MenSCs-Exos, and Exo-free culture supernatant of MenSCs. Estrous cyclicity, ovarian morphology, follicle dynamics, serum hormones, pregnancy outcomes, and molecular changes were investigated.

**Results:**

Exposure to MenSCs-Exos promoted the proliferation of granulosa cells in primordial and primary follicles in vitro and increased the expression of early follicle markers Deleted In Azoospermia Like (DAZL) and Forkhead Box L2 (FOXL2) while inhibiting follicle apoptosis. In vivo, MenSCs-Exos transplantation effectively promoted follicle development in the rat model of POI and restored the estrous cyclicity and serum sex hormone levels, followed by improving the live birth outcome. In addition, transplantation of MenSCs-Exos regulated the composition of the ovarian extracellular matrix and accelerated the recruitment of dormant follicles in the ovarian cortex and increased proliferation of granulosa cells in these follicles.

**Conclusion:**

MenSCs-Exos markedly promoted follicle development in vitro and in vivo and restored fertility in POI rats, suggesting a restorative effect on ovarian functions. The therapeutic effect of MenSCs-Exos transplantation was sustainable, consistent with that of MenSCs transplantation. Our results suggested that MenSCs-Exos transplantation may be a promising cell-free bioresource in the treatment of POI.

## Background

Premature ovarian insufficiency (POI) is defined as the loss of ovarian activity before the age of 40 and is characterized by hormone imbalances, menstrual disorders (amenorrhea or oligomenorrhea), anovulation, and infertility [1]. According to global epidemiological data, POI affects approximately 1% of women younger than age 40, 0.1% of women younger than age 30, and 0.01% of women younger than age 20 [2, 3]. Iatrogenic factors, autoimmune diseases, and genetic abnormalities are the main causes of POI [4, 5].

The clinical features of POI are follicle dysfunction and follicle depletion [6]. The majority of POI patients are diagnosed with diminished ovarian reserve (DOR), which is manifested by the rapid depletion of primordial follicle pool [7]. Between 25 and 50% of POI patients retain menstrual cycles with detectable follicles; however, most of these remnant follicles are dormant. It is difficult for POI patients to restore fertility, and only about 5% of POI patients achieved a clinical pregnancy as result of spontaneous remission [8, 9]. In severe cases, conventional assisted reproductive technology provided a low prognosis unless follicle donation was accepted [10]. In addition, low ovarian response is common in POI patients. Only about 24% of POI patients are able to get ovulatory resumption after hormone supplementation, with a live birth rate of 4%, suggesting that fertile follicles may still be present in some ovaries [11, 12]. Therefore, restoring ovarian reserve and activating dormant follicles are potential strategies for POI treatment.

Recently, transplantation of mesenchymal stem cells (MSCs) was found to be a promising therapy for POI patients. MSCs derived from bone marrow (BMMSCs), umbilical cord (UCMSCs), adipose (ADMSCs), uterine endometrium (EnSCs), and amniotic membrane (AMSCs) have been reported to improve ovarian functions in murine models [13–17]. Our studies using POI mouse models also showed ovulatory improvement and fertility restoration after transplantation of menstrual blood-derived stromal cells (MenSCs) [18]. Recent studies suggested that in situ proliferation and trans-differentiation may not be the main mechanisms through which MSCs improve ovarian function [19–21].

Exosomes (Exos) are extracellular vesicles with a size range between 40 and 150 nm in diameter and could play paracrine roles. By delivering cytoplasmic components of proteins, non-coding RNA, and growth factors, Exos participate in biological processes such as cell proliferation, apoptosis, cytokine production, and immune and metabolic regulation [22]. It has been shown that Exos derived from MSCs have the same efficacy in tissue regeneration and injury restoration as their source cells [23].

Here, we aimed to evaluate whether Exos derived from MenSCs could promote follicular recruitment and development in vitro and investigated the possibility of MenSCs-Exos transplantation in ovulatory restoration in vivo as a cell-free substitute for MenSCs as a strategy for POI treatment.

## Materials and methods

### Ethical approval

This study was approved by the Ethics Committee of the Shengjing Hospital of China Medical University (2020PS461K) and has been performed in accordance with the principles of the Declaration of Helsinki.

### Culture and identification of MenSCs

The culture of MenSCs was consistent with our previous study [24]. Briefly, MenSCs were separated from menstrual blood samples and were cultured in DMEM/F12 medium (1:1, HyClone, Logan, UT, USA) with 10% fetal bovine serum (Gibco, Waltham, MA, USA) under 37 °C with atmosphere containing 5% CO_2_. The culture medium was changed every 3 days. Cells of passage 3 (P3) were collected to identify MSC surface markers of CD44, CD73, CD90, and CD105 using flow cytometry. In this study, P3-P6 cells were selected and labeled with green fluorescent protein (GFP) by lentiviral transfection (MOI 20).

### Extraction and identification of MenSCs-Exos

Briefly, when MenSCs reached 80% confluence, P3-P6 MenSCs were washed twice with PBS and cultured with serum-free DMEM/F12 for 24 h. The supernatant was collected and centrifuged at 2000×*g* for 10 min to remove cell debris and filtered through a 0.22-μm filter. Then, the supernatant was ultracentrifuged at 10,000×*g* for 1 h and 100,000×*g* for 4 h at 4 °C to concentrate exosomes (HITACHI CS120FNX, Japan). Exosomes were labeled with DiI (5 μl/ml, Invitrogen, USA, Cat#C70001) by incubation at 37 °C for 5 min and 4 °C for 15 min before ultracentrifugation at 100,000×*g* for 30 min to remove the un-conjugated dye. The enriched exosomes were diluted in PBS and filtered through a 0.22-μm filter before stored at − 80 °C (named as MenSCs-Exos). The supernatant with exosomes removed was filtered through a 0.22-μm filter and stored at − 80 °C (named as Exo-clear). Transmission electron microscopy (TEM, HITACHI H-7650, Japan) and nanoparticle tracking analysis (NTA) were used to detect the morphology and size of exosomes. Bicinchoninic acid (BCA) protein quantification kit (Beyotime, China, Cat#P0010S) was used to evaluate the protein concentration. Western blot was used to evaluated protein expressions of CD81 and TSG101 (1:1000, Proteintech, China) to identify exosomes.

### Ovarian culture in vitro and analysis

Ovaries from 4.5-day-old female SD rats were harvested and cultured in 8-μm transwell inserts under 37 °C with atmosphere containing 5% CO_2_. The culture medium was 10% FBS-DMEM medium containing 0.05 IU/ml follicle-stimulating hormone (FSH) (LIVZON, China). MenSCs-Exos (2 μg/ml or 20 μg/ml) were added. Ovaries were randomly assigned to receive different treatment (*n* = 5): control, Low-Exos (2 μg/ml), and High-Exos (20 μg/ml). Ovaries were incubated in 300 μl culture medium for 2, 4, and 6 days. The culture medium was changed every 48 h. 1:300 5-ethynyl-2′-deoxyuridine (EdU) solution was added to the medium to detect the proliferation of ovarian cells, before the end of culturing. After EdU incubation for 4 h, the ovaries were collected and fixed in 4% paraformaldehyde. Paraffin sections of 5 μm were prepared. EdU immunofluorescence staining was prepared following the instructions (Riobio, China, C10310). Expressions of DAZL and FOXL2 were evaluated using immunofluorescence using specific antibodies (1:100, Proteintech). Cell apoptosis was detected using TUNEL staining (*TransDetect*® In Situ Fluorescein TUNEL Cell Apoptosis Detection Kit, FA201-01) and evaluated by using Nikon Eclipse Ni (Nikon, Tokyo, Japan) and Image Pro Plus 6.

### Establishment and treatment of the POI rat model

SD rats were purchased from HFK Bioscience Co. (Beijing, China) and housed in an SPF (specific pathogen-free) lab (SYXK 2017-0004, China) in an environment with a temperature of 22 ± 1 °C, relative humidity of 50 ± 1%, and a light/dark cycle of 12/12 h. Sterilized food and water were available ad libitum. All animal studies (including the euthanasia procedure) were performed in compliance with the regulations and guidelines of China Medical University institutional animal care and conducted according to the AAALAC and the IACUC guidelines.

The chemical compound VCD (4-vinylcyclohexene diepoxide, Merk Millipore) was intraperitoneally injected into female SD rats with regular estrous cycles (7 weeks old, body weight 180–200 g) for 15 consecutive days (80 mg/kg per day). Vaginal smears for 7 consecutive days were taken from the 8th day of injection to confirm the modeling effect. After that, forty-eight modeled rats were randomly signed into four groups: PBS injection group (control group), 5 × 10^5^ MenSCs per ovary suspended in PBS injection group (MenSCs group,), 25 μg MenSCs-Exos per ovary suspended in PBS injection group (MenSCs-Exos group), and exosome-removed MenSC culture supernatant injection group (Exo-clear group). The received fluid amount was 50 μl per ovary, and intra-ovary injection was performed with a 32G syringe. Every 5 days, all rats received caudal intravenous injection of 100 μl: PBS for control and MenSCs group, 50 μg exosomes for MenSCs-Exos group, and exosome-removed MenSC culture supernatant for Exo-clear group. Daily vaginal smears were performed from the 7th day of treatment for 21 days. After 28 days of treatments, eight rats of each group were sacrificed at the first diestrous phase and then the ovaries and serum samples were harvested. The main organs (brain, heart, lung, liver, spleen, kidney, uterus, and ovary) of MenSCs and MenSCs-Exos group were used for bioluminescence imaging (BLI) using the in vivo MS FX Pro system (Carestream, USA) and analyzed by the Carestream MI SE software. After removing the surrounding fat pads and fallopian tubes, ovary weights and ovary/body weight ratios were evaluated (ovary ratio = ovary weight/body weight × 100%). Then, the left ovary was fixed for histology and the right ovary and serum samples were stored at − 80 °C. Eight healthy female SD rats were assigned as the normal group.

### ELISA

Serum anti-Mullerian hormone (AMH), follicle-stimulating hormone (FSH), and estradiol (E2) levels were monitored according to the standard method of ELISA kit (CUSABIO, China). Ninety-six-well plates with antibodies were incubated with 1:10 diluting serum, and the reaction was conducted at room temperature for 2 h (*n* = 8). Absorbance was recorded by using a microplate reader, to determine serum hormone concentration.

### Evaluation of reproductive potential

After 28 days of treatment, 4 rats in each group were randomly selected to mate with healthy male SD rats (1:2). The second morning, sperm plugs represented successful mating. Each female rat was mated again after delivery and lactation (3 weeks postpartum). The number of cubs of the two births was recorded. Female rats that were sperm positive for three consecutive times but did not give birth were judged to be infertile.

### Histological analysis

Rat ovaries were fixed with 4% paraformaldehyde, dehydrated, and embedded in paraffin. A series of 5-μm sections were prepared and one of every five sections was selected for hematoxylin and eosin staining (H&E). Ovarian morphology was observed and photographed with an optical microscope (Nikon 80i). The total number of primordial follicles, primary follicles, secondary follicles, antral follicles, and preovulatory follicles were counted.

### Real-time PCR

The extraction of ovarian RNA and RT-PCR analysis were described by us previously [24]. Briefly, total RNA was extracted from tissues by using the RNAiso Plus kit (Takara, China, Cat#9108) before reverse transcribed to generate cDNA (Takara, China, Cat#RR047A). Quantitative real-time PCR using 96-well optical plates was performed in a SYBR Green (Takara, China, Cat#RR820A) format with 7500 Fast Real-Time PCR Detection Systems (Life Technology); each sample was done in triplicates. We detected the genes related to apoptosis of *Bcl2* (Bcl2, apoptosis regulator), *Bad* (Bcl2 associated agonist of cell death), *Bax* (Bcl2 associated X), and *Casp8* (Caspase 8) between the control group and low-Exos group after 2 days of in vitro culturing (*n* = 6). We also evaluated the gene expression of follicles development: *Zp3* (zona pellucida glycoprotein 3), *Th* (tyrosine hydroxylase), *Amh* (anti-Mullerian hormone), and *Fshr* (follicle stimulating hormone receptor) in POI ovaries after treatments (*n* ≥ 6). Primer sequences used for each target gene are summarized in Additional file 1: Table S1. Analyses of relative gene expressions were performed using 2^−ΔΔCT^ methods. Ratios of mRNA expression were given as fold-changes relative to untreated controls after normalizing to allogeneic *Gapdh* housekeeping gene.

### Immunohistochemistry and immunofluorescence

Ovarian paraffin sections were dewaxed with xylene and rehydrated with alcohol gradient before immunohistochemistry and immunofluorescence. Following the instructions of the immunohistochemical kit (SP-9000, ZSGB-BIO, China), after the sodium citrate solution heating and repairing (ZLI-9064 pH 6.0, ZSGB-BIO, China), all slices were sealed with H_2_O_2_ and 10% goat serum. The sections were then incubated with the first antibody at 4 °C overnight (Ki-67, Collagen 1 and FOXL2,1:100, Proteintech, China; Collagen IV, FN1 and Laminin,1:100, Bioss, China). On the second day, all sections were washed with PBS and incubated with the secondary antibody for 2 h at room temperature. The primary antibody was replaced by PBS as a negative control. The detection of bound antibody was performed using avidin-biotin complex. DAB was performed at room temperature for 1 min as chromogen. The nuclei were counterstained with Mayer’s hematoxylin for 2 min. Staining intensity was recorded as 0 (negative), 1 (weak), 2 (medium), and 3 (strong). The positive cells were observed and evaluated using an optical microscope (Nikon 80i).

### Statistical analysis

One-way analysis of variance (ANOVA) was used to analyze variables among groups, and Bonferroni post hoc tests were performed for multiple comparisons when statistical significance was recognized. All numerical values are presented as the mean ± standard deviation (SD), and *P < 0.05, **P < 0.01, and ***P < 0.001 were considered to indicate a statistically significant difference. All statistical analyses were performed using GraphPad Prism 8 software (San Diego, CA, USA).

## Results

### Characterization of MenSCs and MenSCs-Exos

**Fig. 1:**
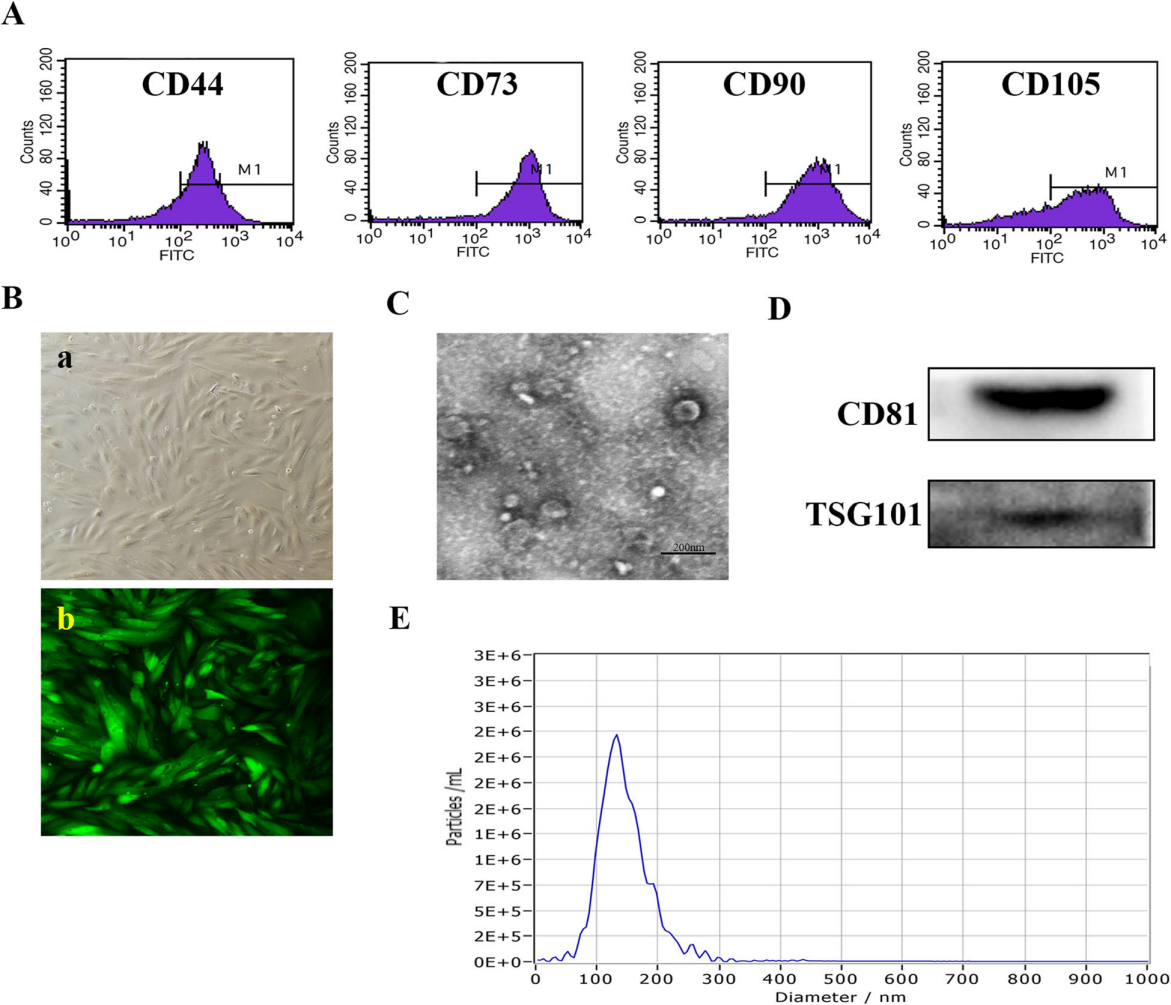
Characterization of MenSCs and MenSCs-Exos. **a** Surface markers of MenSCs detected by Flow cytometry. **b** Morphology of P3 MenSCs and GFP-labeled MenSCs (200×). **c** Morphology of MenSCs-Exos observed by TEM (scale bar = 200 nm). **d** Western blots of CD81 and TSG101 of MenSCs-Exos. **e** Size distribution of MenSCs-Exos examined by NTA

As our previous studies described [24–26], MenSCs had a spindle shape, fibroblast-like morphology, and tested positive for CD44, CD73, CD90, and CD105 (Fig. 1A, B). Exosomes isolated from serum-free culture supernatant of MenSCs were characterized by TEM, NTA, and western blotting. MenSCs-Exos exhibited a sphere-shaped morphology with a concentration of 1.1 × 10^10^ particles/ml and a peak diameter of 128 nm. BCA analysis showed that the protein concentration of MenSCs-Exos was 1.75 mg/ml. MenSCs-Exos were confirmed to be positive for CD81 and TSG101 by Western blot analysis (Fig. 1C–E).

### MenSCs-Exos treatment promoted early follicular development and inhibited apoptosis in vitro

**Fig. 2:**
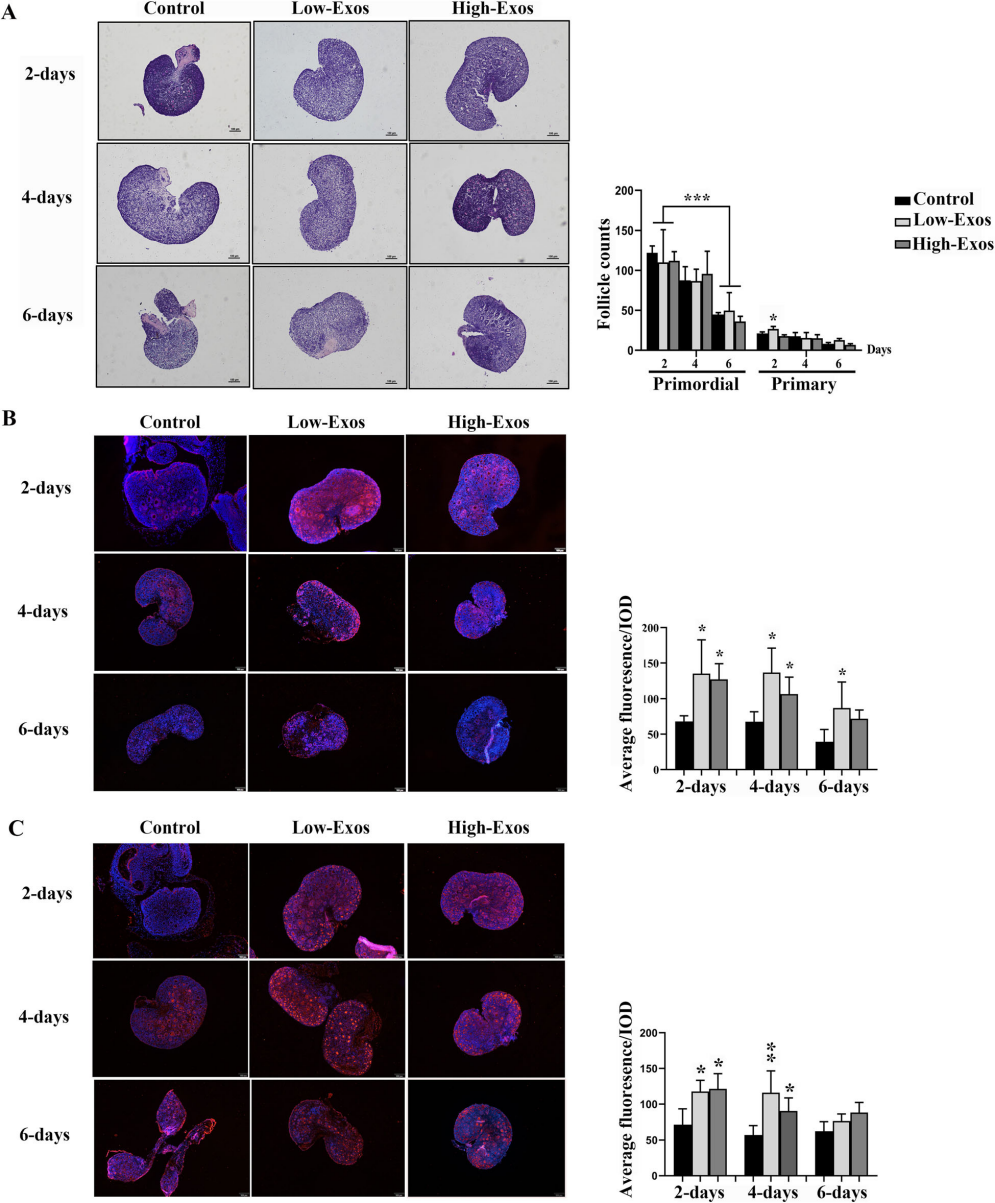
Ovarian morphology and immunofluorescence analysis of rat ovaries cultured in vitro. **a** H&E staining of ovaries sections (× 100) and follicles counts. **b** Immunofluorescence of DAZL in ovaries (red) and statistics of fluorescence intensity. Nuclei were stained by using DAPI (blue, 100×, *n* ≥ 4). **c** Immunofluorescence of FOXL2 of ovaries and statistics of fluorescence intensity. Nuclei were stained by using DAPI (blue, × 100, *n* ≥ 4). Data were represented as mean ± SD, *p < 0.05, **p < 0.01, ***p < 0.001

The ovaries of 4.5-day-old rats were cultured in culture medium containing 0, 2 μg/ml (low-Exos), or 20 μg/ml (high-Exos) of MenSCs-Exos for 2, 4, and 6 days. With the extension of culture time, there was significant decrease in the number of primordial follicles with nuclei in the ovaries of all groups. At the same time, atretic follicles were observed. Low-Exos treatment for 2 days significantly increased the number of primary follicles in ovaries cultured in vitro (Fig. 2a). Next, we evaluated follicle markers in the 3 groups. Compared to the control group, Low-Exo treatment for 2–6 days, and High-Exo treatment for 2 and 4 days, significantly increased the DAZL immunofluorescence intensity in the ovaries cultured in vitro (Fig. 2b). In addition, after 2 and 4 days of treatment, a significant increase in the expression of FOXL2, a marker for granulosa cells, was detected in the ovaries of the Low-Exos and High-Exos groups (Fig. 2c).

**Fig. 3:**
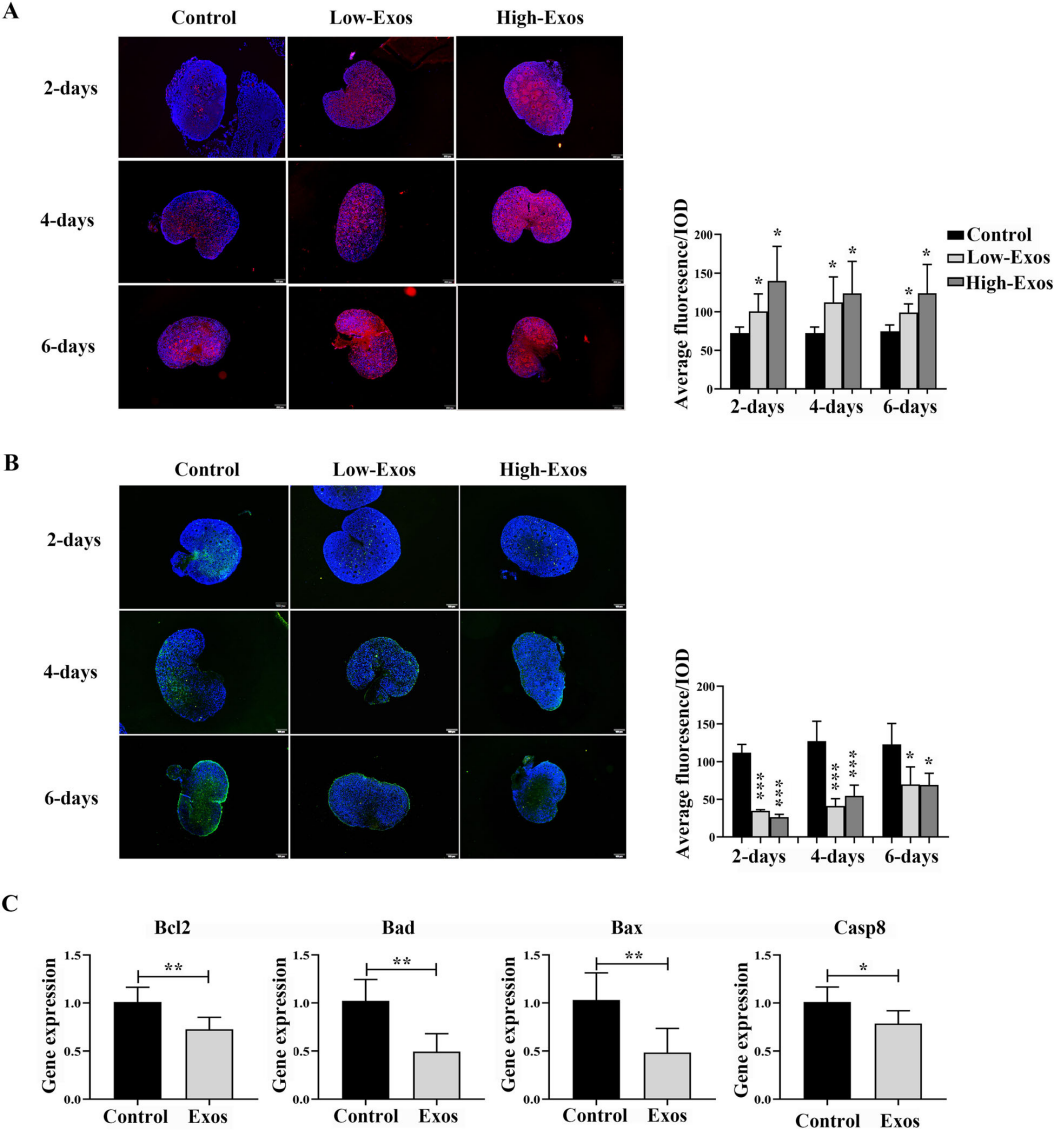
Cell proliferation and apoptosis of rat ovaries cultured in vitro. **a** EdU staining of ovaries (red) and statistics of fluorescence intensity. Nuclei were stained by using DAPI (blue, × 100, *n* ≥ 4). **b** TUNEL staining of apoptosis (green) and statistics of fluorescence intensity. Nuclei were stained by using DAPI (blue, × 100, *n* ≥ 4). **c** RT-PCR analysis of *Bcl2*, *Bad*, *Bax*, and *Casp8* in ovaries (*n* = 6). Data were represented as mean ± SD, *p < 0.05, **p < 0.01, ***p < 0.001

EdU-positive cells, as indicated by the high fluorescence intensity, were detected in both the Low-Exos and High-Exos groups after 2, 4, and 6 days of culture (Fig. 3a). With the extension of culture time, we observed that in the ovaries of all groups, TUNEL-positive cells increased from the center of the ovary with some follicles being TUNEL-positive. A significant decrease in TUNEL fluorescence intensity levels was detected in both the Low-Exos and High-Exos groups during the entire ovarian culture experiment (Fig. 3b). There was no difference between the Low-Exos and High-Exos groups with respect to either EdU- or TUNEL-positive fluorescence intensity. In addition, we examined the expression of apoptosis-related genes in the control and Low-Exos groups after 2 days of in vitro incubation (*n* = 6). The results indicated that MenSCs-Exos treatment for 2 days significantly downregulated the expression of *Bcl2* (*p =* 0.0059), *Bad* (*p* = 0.0013), *Bax* (*p* = 0.0054), and *Casp8* (*p* = 0.0302) (Fig. 3c). The above results were consistent with the TUNEL staining. These results indicated that MenSCs-Exos could act on the early stage of oocytes, promote follicular development and granulosa cell proliferation, and inhibited tissue apoptosis.

### MenSCs-Exos showed tropism toward the ovaries of POI rats

**Fig. 4:**
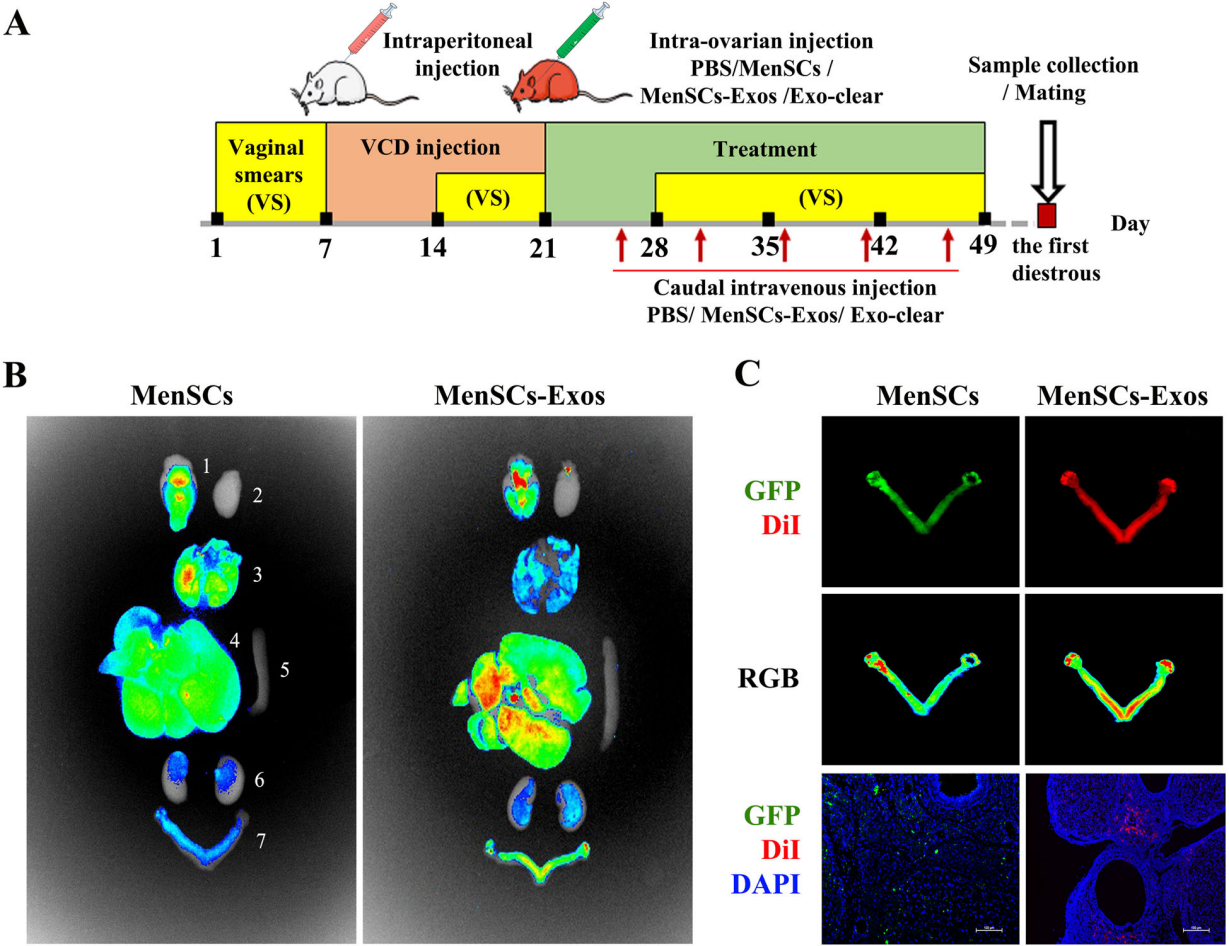
Experimental procedure chart and locations of transplants in MenSCs and MenSCs-Exos group. **a** Scheme of this experiment. **b** Fluorescence intensity (RGB) in main organs ((1) brain, (2) heart, (3) lung, (4) liver, (5) spleen, (6) kidney, (7) genital system; blue, low fluorescence intensity, green, moderate fluorescence intensity, red, high fluorescence intensity). **c** Fluorescence localization in genital system and in frozen sections (green, GFP, red, DiI, blue, DAPI, × 100)

Scheme of in vivo experiments was shown in Fig. 4a. Briefly, we administered VCD intraperitoneally to SD rats (with regular motility cycles) for 15 consecutive days to establish the POI phenotype. After confirming the irregular cycle, 48 POI rats were randomly grouped for a single intra-ovarian injection treatment. Subsequently for every 5 days, rats in the MenSCs-Exos group received exosomes, the Exo-clear group received exosome-removed MenSC culture supernatant and other rats received PBS via caudal intravenous injection. Vaginal smear was observed continuously for 28 days during treatment. At the first diestrous post-treatment, 8 rats in each group were sacrificed and 4 rats were caged with males to observe two-times fertility outcomes.

To trace the location of the transplanted cells and exosomes, MenSCs were transfected with GFP and MenSCs-Exos were labeled with DiI. According to the results of BLI, GFP intensity was strong in the basal ganglia and lungs; moderate in the liver; weak in the ovaries, uterus, and kidneys; and non-existent in the heart and spleen after 28 days of treatment. As for the fluorescence of DiI, the intensity was strong in the liver and basal ganglia, moderate in ovaries and uteri, weak in the lungs and kidneys, and non-existent in the heart and spleen (Fig. 4b). A strong GFP signal was detected in the bilateral ovarian cortex. The DiI signal was detected in the bilateral ovarian cortex and the lower uterine segment. It was observed that MenSCs and MenSCs-Exos were integrated in the somatic cells of the ovarian cortex using immunofluorescence (Fig. 4c). These results indicated that both MenSCs and MenSCs-Exos showed tropism toward the ovarian cortex in POI rats.

### MenSCs-Exo treatment restored the estrous cyclicity in POI rats in vivo

**Fig. 5:**
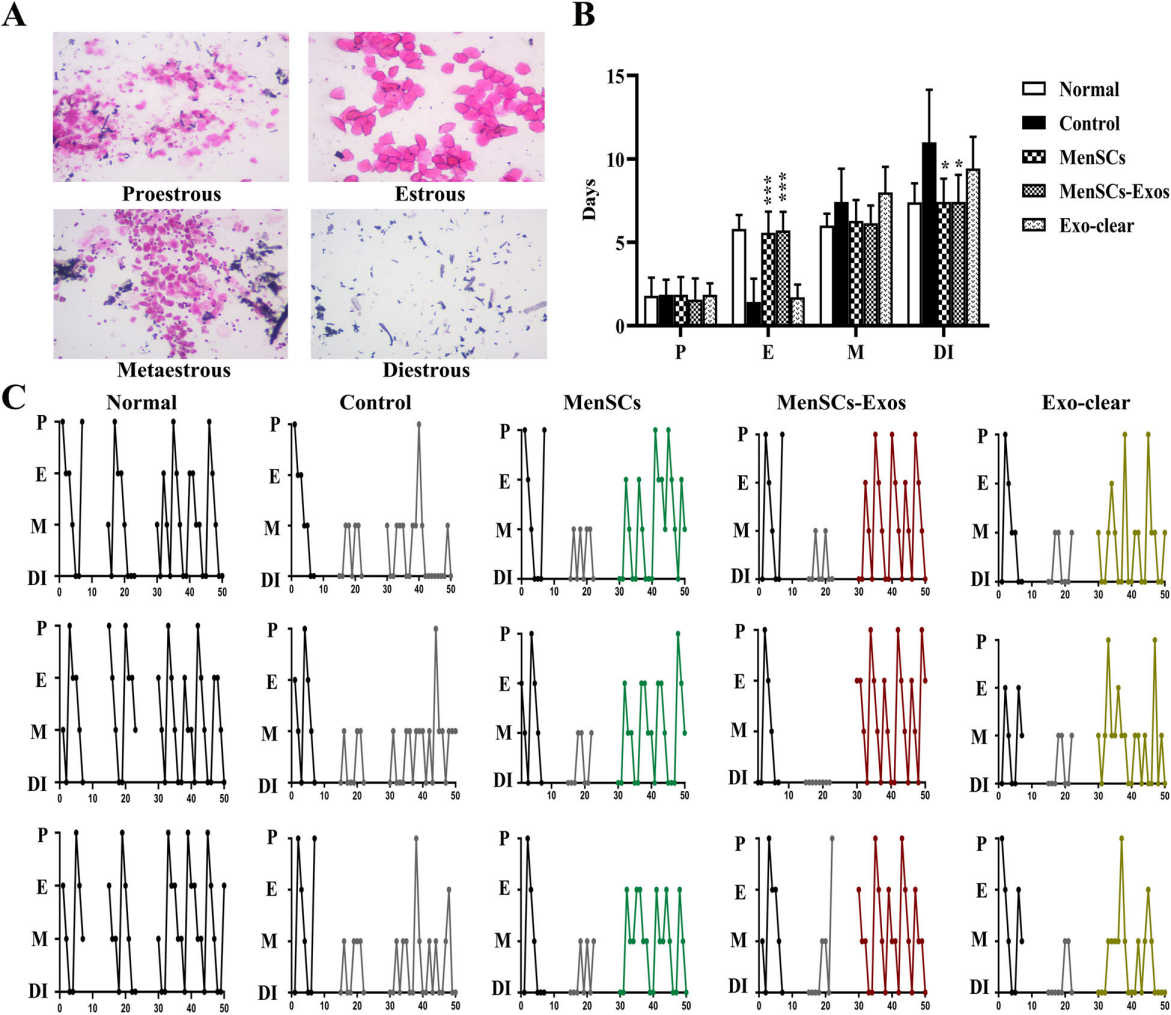
Statistics of estrous cycle in different group of rats. **a** H&E staining of vaginal smears (× 100×). **b** Compositions of estrous cycle in different groups (*n* = 8; P, proestrous; E, estrous; M, metaestrous; DI, diestrous). **c** Typical estrous cycle patterns in normal rats and in four treatment groups. Data were represented as mean ± SD, *p < 0.05, **p < 0.01, ***p < 0.001

We compared the stages of the estrous cycle in 4 treatment groups and the control group. Regular estrous cycles were founded in control female rats, showing a stable pattern. In the control group, the diestrus stage (DI stage) was predominant, followed by metestrus stage (M stage), almost without proestrus stage (P stage), and estrus stage (E stage). Compared with the control group, the frequency of E stage was significantly increased in the MenSCs and MenSCs-Exos groups (*P*_MenSCs_ < 0.0001, *P*_MenSCs-Exos_ < 0.0001), and the DI stage was significantly decreased (*P*_MenSCs_ = 0.0250, *P*_MenSCs-Exos_ = 0.0262). There was no statistical difference between the Exo-clear group (Fig. 5b) and the control. After VCD injection, it was observed that the estrous cycle of SD rats mainly changed into the M stage and the DI stage. The rats in the control group and the Exo-clear group had irregular estrus cycles after treatment. After MenSC transplantation, regular estrous cycles were restored in 11 rats, and a total of 40 cycles were observed with an average of 4.85 days. All rats in the MenSCs-Exos group showed restored regular estrous cycles, and 45 cycles were observed with an average of 4.44 days. Four stages were observed in the Exo-clear group but without a regular pattern (Fig. 5c).

### MenSCs-Exos improved ovarian morphology, increased follicle numbers, regulated serum hormones, and restored fertility in POI rats in vivo

**Fig. 6:**
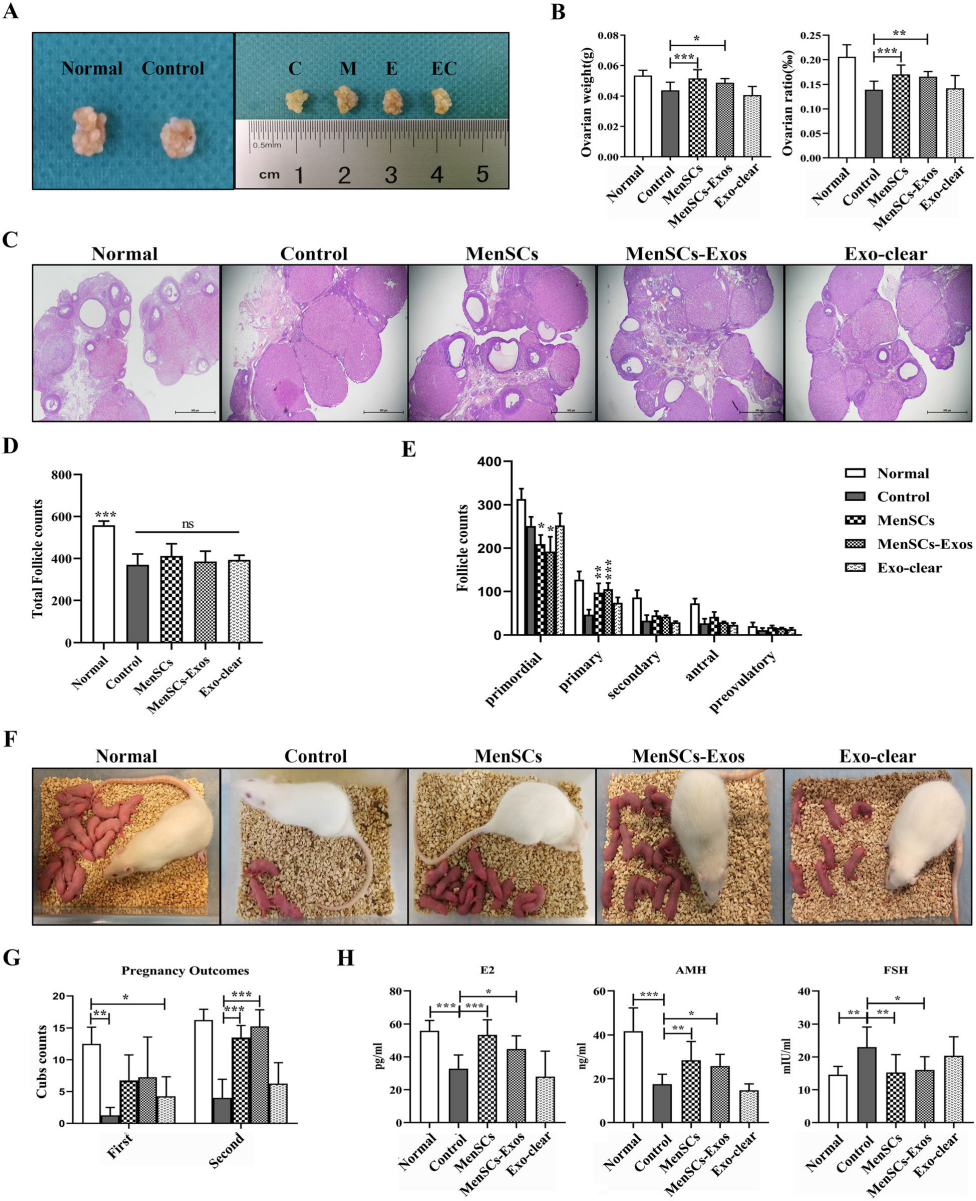
Ovarian morphological, ELISA assays, and live birth outcomes after treatments. **a** Representative ovarian morphology ((C) control group, (M) MenSCs group, (E) MenSCs-Exos group, (EC) Exo-clear group). **b** Ovarian weight (left) and ovarian/body weight ratio (right). **c** H&E staining of ovarian sections (× 40). **d** Total follicle counts. **e** Counting of follicles at different stages. **f** Live births photos of normal rats and four treatment groups. **g** Live births counts. **h** Serum levels for E2 (left), AMH (middle), and FSH (right) (*n* = 8). Data were represented as mean ± SD, *p < 0.05, **p < 0.01, ***p < 0.001

VCD treatment decreased ovarian sizes whereas ovarian sizes of POI model rats increased after receiving MenSCs or MenSCs-Exos transplantation (Fig. 6a). Compared to the control group, the ovarian weight (*P*_MenSCs_ = 0.0008, *P*_MenSCs-Exos_ = 0.0054) and ovarian ratios (*P*_MenSCs_ = 0.0001, *P*_MenSCs-Exos_ < 0.0001) were significantly increased in both the MenSCs and MenSCs-Exos groups, but not in the Exo-clear group (Fig. 6b).

Histological analyses indicated that VCD treatment led to ovarian atrophy, mainly consisting of corpora lutea. In the MenSCs and MenSCs-Exos groups, there were a number of primordial, primary, secondary, and antral follicles together with corpora lutea. In contrast, only a few follicles were observed in the Exo-clear group (Fig. 6c). The total number of follicles in all POI ovaries decreased significantly when compared with the normal group, and there was no difference in the total number of follicles between the four treatment groups (Fig. 6d). Compared to the control group, after MenSCs and MenSCs-Exos transplantation, the number of primordial follicles significantly decreased while the number of primary follicles increased markedly. There was no difference in the number of secondary follicles, antral follicles, and preovulatory follicles in each group (Fig. 6e).

The fertility of POI rats was assessed with consecutive mating experiments. All females were mated again at the end of the lactation period. After the first mating, an increasing trend in the number of live births was observed in the MenSCs and MenSCs-Exos groups, but without statistical significance. The second litter size was greater than the first, for all groups. After the second mating, the number of births in the MenSCs and MenSCs-Exos groups increased significantly (*P*_MenSCs_ = 0.02, *P*_MenSCs-Exos_ = 0.005), when compared to the control group, but not in the Exo-clear group (Fig. 6f, g).

Next, we evaluated the serum concentrations of E2, AMH, and FSH at the DI stage. Compared to non-treated rats, serum E2 and AMH levels in POI rats were significantly decreased (*p* < 0.001) and the FSH level was increased (*p* = 0.002). After the MenSC and MenSCs-Exo treatments, serum E2 and AMH levels in POI rats significantly increased (*P*_MenSCs_ < 0.001 and *P*_MenSCs-Exos_ = 0.03 for E2, *P*_MenSCs_ = 0.001 and *P*_MenSCs-Exos_ = 0.03 for AMH) while the FSH level significantly decreased (*P*_MenSCs_ = 0.004 and *P*_MenSCs-Exos_ = 0.02). There was no difference in the levels of these hormones between the MenSCs group and the MenSCs-Exos group (Fig. 6h).

These results indicated that MenSCs-Exos treatment improved the ovarian atrophy caused by VCD, effectively restoring estrous cyclicity and promoting follicle development. Multiple transplantation of MenSCs-Exos had a restorative effect on the female sex hormones, thus improving fertility in POI rats, and achieving an equivalent therapeutic effect similar to single intra-ovarian transplantation of MenSCs.

### MenSCs-Exos promoted follicle development and increased the proliferation of granulosa cells in vivo

**Fig. 7:**
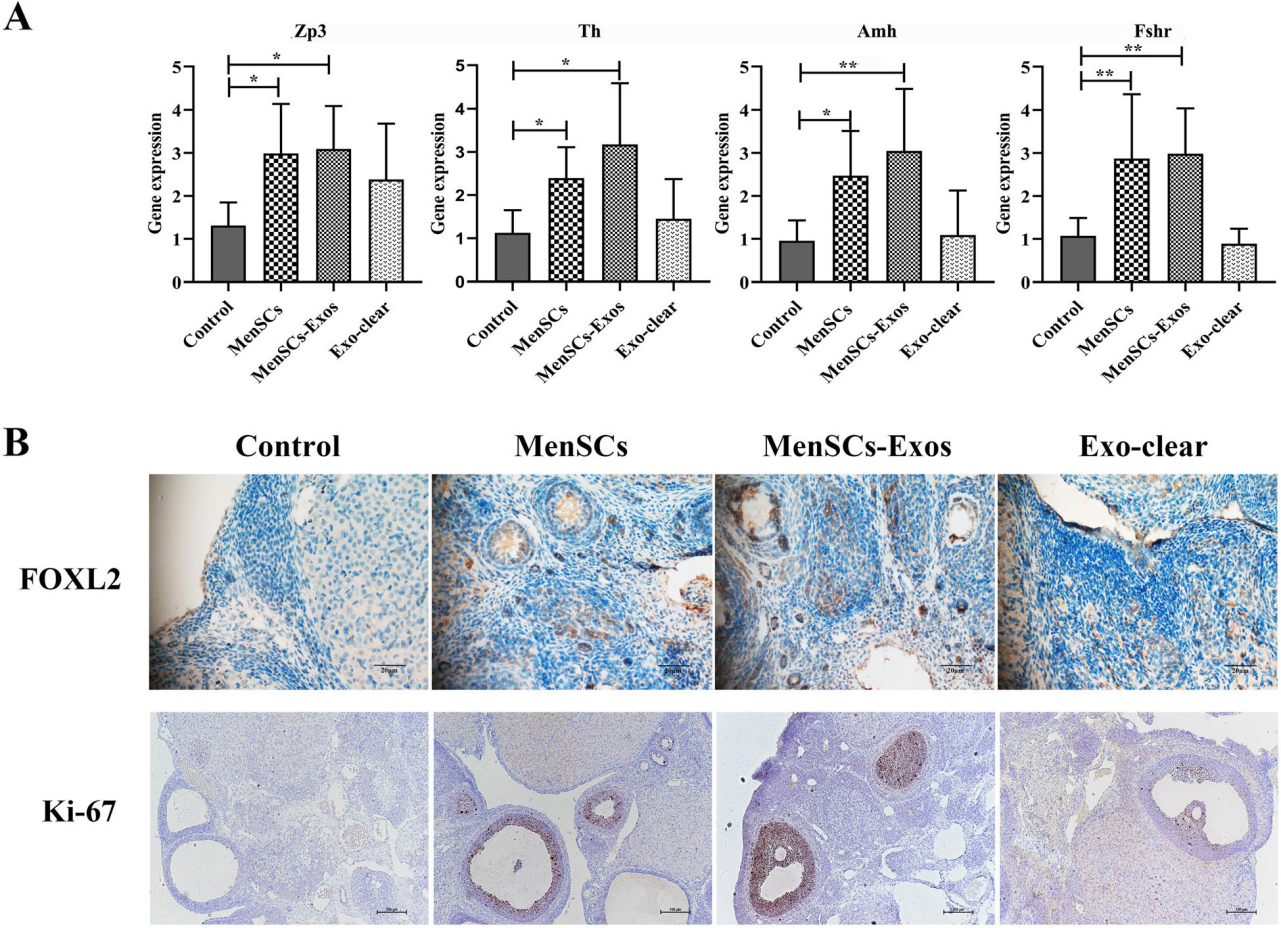
Real-time PCR and immunohistochemistry analysis of follicle development in four groups. **a** RT-PCR analysis of *Zp3*, *Th*, *Amh,* and *Fshr* in ovaries (*n* ≥ 6). **b** Typical IHC staining of FOXL2 (× 400) and Ki-67 (× 100), *n* ≥ 4

Ovarian gene expression was evaluated by RT-PCR, immunofluorescence, and immunochemistry. As evaluated by RT-PCR, *Zp3* (*P*_MenSCs_ = 0.04, *P*_MenSCs-Exos_ = 0.02), *Th* (*P*_MenSCs_ = 0.03, *P*_MenSCs-Exos_ = 0.04), *Amh* (*P*_MenSCs_ = 0.03, *P*_MenSCs-Exos_ = 0.004), and *Fshr* (*P*_MenSCs_ = 0.01, *P*_MenSCs-Exos_ = 0.005) were significantly upregulated after MenSCs and MenSCs-Exos transplantation (Fig. 7a). Compared to the control and Exo-clear groups, there was a significant expression of FOXL2 detected in the single-layer granulosa cells of primordial follicles after the MenSCs and MenSCs-Exos treatments. The expression of Ki-67 was mainly detected in the multiple layers of granulosa cells of follicles. An increase in the levels of Ki-67-positive cells was found in the MenSCs and MenSCs-Exos groups when compared with the control group. Moreover, Ki-67-positive cells were also observed in the ovarian stroma (Fig. 7b).

### MenSCs-Exos regulated the ovarian extracellular matrix (ECM) in POI

**Fig. 8:**
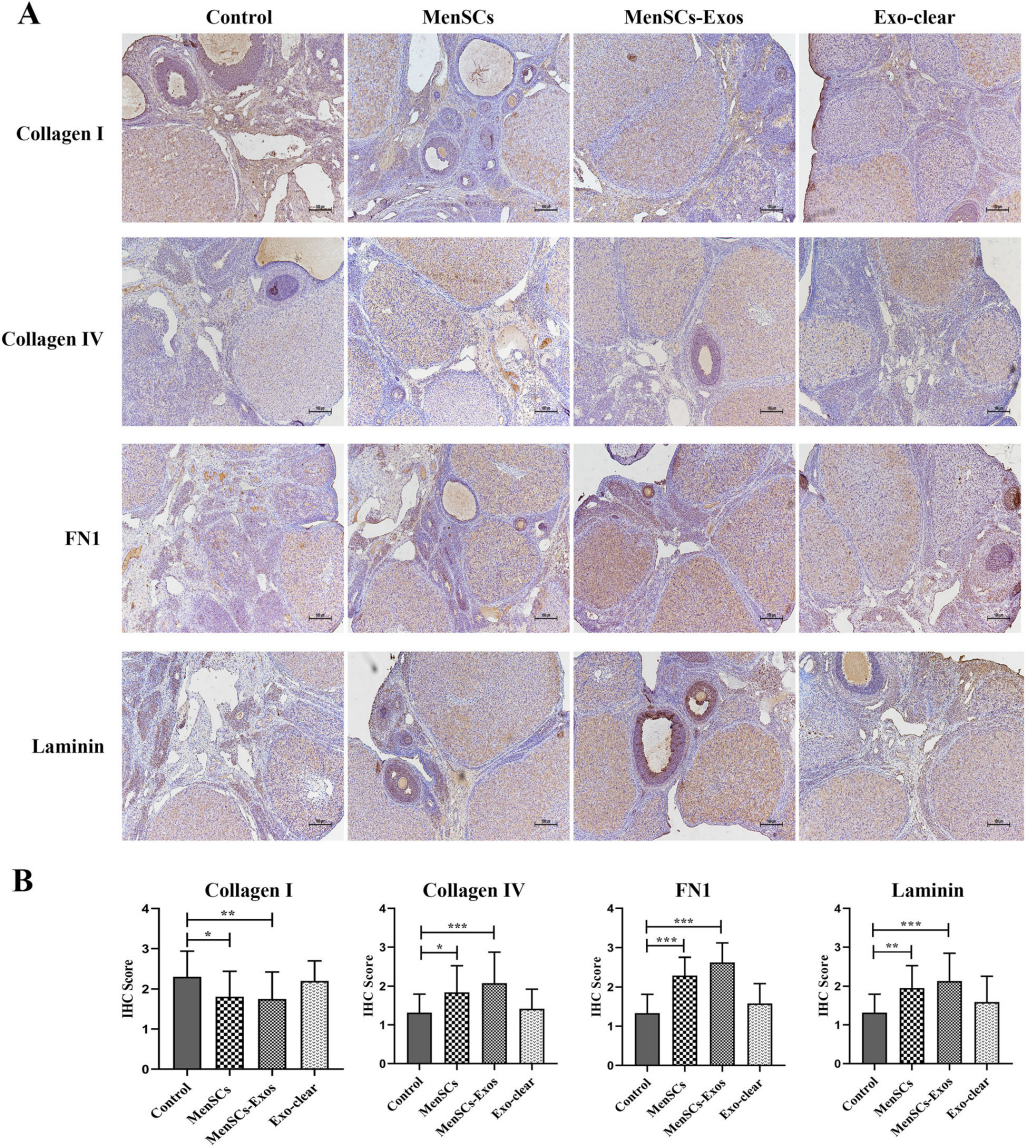
Immunohistochemistry of ovarian ECM components in four groups. **a** Typical IHC staining of Collagen I, Collagen IV, FN 1, and Laminin (× 100). **b** IHC scores. Data were represented as mean ± SD, *p < 0.05, **p < 0.01, ***p < 0.001

ECM components were analyzed by immunohistochemistry. Compared to the control group, there was a significant decrease in the expression of Collagen I in the POI ovaries after MenSCs and MenSCs-Exos transplantation. In the MenSCs and MenSCs-Exos groups, the expression of Collagen IV, FN1, and Laminin significantly increased in the follicular basement membrane and ovarian stroma of the cortex. The increased expression of FN1 and Laminin was also observed in the granulosa cells. There was no statistical difference between the Exo-clear group and the control group. The above results revealed that MenSCs-Exos regulated the ECM components of POI ovaries and had the same effect as MenSCs (Fig. 8a, b).

## Discussion

This study demonstrated that MenSCs-Exos treatment is effective in restoring ovarian functions and fertility in a rodent model of POI. Short-term incubation with MenSCs-Exos enhanced the growth and development of early stage follicles during ovarian culture in vitro, while inhibiting the apoptosis of ovarian cells. In the POI rat model, repeated applications of MenSCs-Exos reversed ovarian insufficiency induced by a chemo-toxic drug, markedly promoted follicular development, and restored hormonal profiles, ovarian ECM remodeling, and fertility in vivo. The therapeutic effect of repeated applications of MenSCs-Exos was comparable to that of MenSC transplantation, suggesting a promising biological alternative to the use of cell-free bioresource for the restoration of ovarian function.

Over the last decade, MSC transplantation has been shown to be effective in the repair of systemic injuries such as in the motor system, nervous system, digestive system, and more specific reproductive system [27]. MenSCs are endometrial stem cells isolated from menstrual blood shedding with typical MSC characteristics. In addition to having similar functions and mechanisms to common sources of MSCs (BDMSCs, ADMSCs, and UCMSCs), MenSCs’ periodic non-invasive acquisition and ability to be used for autologous transplantation highlights their great potential for clinical applications [28]. In our rodent study, we demonstrated that MenSCs effectively restored ovulation and fertility in a POI murine model [18]. Although MSC application is widely considered to be safe and almost nontumorigenic, the risk of contamination and embolization should not be overlooked [29, 30]. It is worth noting that the therapeutic effects of stem cell preparations or derivatives have gradually been confirmed. Several studies suggested that the culture medium of MSCs (MSC-CM) could have therapeutic effects in POI murine models. Promotions of follicular number, ovarian angiogenesis, and decrease of granulosa cell apoptosis were detected after MSC-CM application, suggesting a paracrine effect of MSCs in ovarian treatment [31, 32]. Compared to MSC transplantation, Exos are biocompatible and non-immunogenic, thus allowing allo-transplantation. Therefore, Exos treatment may be a promising cell-free therapy in regenerative medicine. In addition, the application of Exos can be personalized which makes MSC-Exo-based therapy a safer and more effective treatment than MSC transplantation [33]. Exos derived from ADMSCs and AMSCs that were demonstrated to restore ovulatory function in a POI mouse model, mainly acted on granulosa cells [34, 35]. Recently, UCMSCs-Exos were found to improve oocyte production and quality in aged female mice in vivo and accumulated primordial follicles in vitro, suggesting a new approach of mitigating fertility retardation [36]. Similarly, we demonstrated therapeutic effects of MenSCs-Exos in restoring ovarian function in rodent POI model.

In this study, we established a rat model of POI by intraperitoneal injection of VCD. VCD is a chemical compound causing small preantral (primordial and primary) follicle degeneration in rats [37]. Therapeutically, gonadotropin treatments mainly act on growing follicles, while the remaining dormant primordial follicles are not affected in the POI ovaries. For POI patients, in vitro activation (IVA) was effective by following in vitro treatment of ovarian cortical pieces with Akt-stimulating drugs to activate dormant primordial follicles and then re-transplanted under the serous membrane of the fallopian tube [38]. Recently, IVA application without the use of PTEN/PI3K/Akt signaling modulators has been demonstrated effective in follicular activation [39]. However, the operational procedures and equipment requirements remain as challenges for the clinical application of IVA. Our recent results indicated the benefits of MenSCs-Exos in ovarian culture in vitro. In this study, we found the promotion of primordial follicular activation and development by using cell-free MenSCs-Exos, as well as inhibition of cell apoptosis during ovarian culture in vitro.

The successful maturation of oocytes is associated with hormone regulation, granulosa cell function, corpus luteum development, and ovarian microenvironment [40]. Li et al. suggested that the synchronous development of oocytes and ovarian cells plays a key role in follicular formation [41]. Increased FOXL2 expression was also detected in the MenSCs and MenSCs-Exos groups. As a marker of pre-granulosa cells, FOXL2 is essential for the functional maintenance of follicle development. An absent expression of FOXL2 is suggested to be related with abnormal structure and activity of granulosa cells, resulting in follicular formation failure [42, 43]. The ovarian stroma has been shown to play a key role in follicular development. In the cortex of the adult ovary, there is a continuous basal layer between the ovarian stroma, corpus luteum, follicle, and the ovarian epithelium. These basal layers generally consisted of laminin, collagen, and fibronectin, which constitute the follicular ECM. In addition, follicular ECM is believed to be involved in maintaining follicular growth, which is considered a part of the follicular microenvironment [44]. In this study, we found that MenSCs-Exos enhanced the expression of Laminin, Collagen IV, and FN1 in POI ovaries, suggesting an effect on ovarian microenvironment regulation. In 2019, Macdonald et al. indicated that the addition of Collagen IV promoted meiosis and follicular formation in vitro. Laminin is suggested as an important ECM component for human follicular development. Secreted by oocytes, Laminin has been proved to promote ovarian stem cell differentiation in vitro [45].

There are some limitations in the present study that need to be pointed out: (1) In this study, it was preliminarily confirmed that MenSCs-Exos promoted both oocytes and granulosa cells. Yet follicular maturation is dependent on the interaction between oocyte and granulosa cells. Further explorations of MenSCs-Exos effects on oocyte-granulosa cross-talking or gap-junction are necessary. (2) The molecular mechanism involved in MenSCs-Exos needs to be further explored. Identifying the active components of MenSCs-Exos for improving ovarian function (such as protein or micro-RNA) is conducive to improving the efficiency of MenSCs-Exos in the treatment of POI.

## Conclusions

In summary, we confirmed that MenSCs-Exos treatments were beneficial in vitro and had a significant therapeutic effect in restoring ovarian functions in a rat POI model. Ovarian reserve, serum hormones, and fertility were all improved after MenSCs-Exo transplantation. However, the molecular mechanisms by which MenSCs-Exos restore ovarian function remain to be further explored. Overall, our results suggested that MenSC-Exos transplantation may be a promising cell-free bioresource in the treatment of POI.

## Supplementary Information

**Additional file 1: Table S1.** Quantitative polymerase chain reaction primer sequences.

## Data Availability

The data that support the findings of this study are available from the corresponding author upon reasonable request.

## Abbreviations

POI: Premature ovarian insufficiency
MenSCs: Menstrual blood-derived stromal cells
MenSCs-Exos: MenSCs-derived exosomes
SD rats: Sprague Dawley rats
VCD: 4-Vinylcyclohexene diepoxide
DAZL: Deleted In Azoospermia Like
FOXL2: Forkhead Box L2
DOR: Diminished ovarian reserve
MSCs: Mesenchymal stem cells
BMMSCs: Bone marrow-derived stem cells
UCMSCs: Umbilical-derived stem cells
ADMSCs: Adipose-derived stem cells
EnSCs: Endometrium-derived stem cells
AMSCs: Amniotic membrane-derived stem cells
Exos: Exosomes
P3: Passage 3
P6: Passage 6
GFP: Green fluorescent protein
PBS: Phosphate-buffered saline
TEM: Transmission electron microscopy
NTA: Nanoparticle tracking analysis
BCA: Bicinchoninic acid
FSH: Follicle-stimulating hormone
EdU: 5-Ethynyl-2′-deoxyuridine
SPF: Specific pathogen-free
BLI: Bioluminescence imaging
AMH: Anti-Mullerian hormone
E2: Estradiol
H&E: Hematoxylin and eosin
ELISA: Enzyme-linked immunosorbent assay
Bcl2: Bcl2, apoptosis regulator
Bad: Bcl2-associated agonist of cell death
Bax: Bcl2-associated X
Casp8: Caspase 8
Zp3: Zona pellucida glycoprotein 3
Th: Tyrosine hydroxylase
Fshr: Follicle-stimulating hormone receptor
ECM: Extracellular matrix
ANOVA: One-way analysis of variance
SD: Standard deviation
