# Supplementary material for: Concentrated exosomes from menstrual blood-derived stromal cells improves ovarian activity in a rat model of premature ovarian insufficiency
Source: Stem Cell Res Ther. 2021 Mar 12;12:178. doi: 10.1186/s13287-021-02255-3 (PMC7953711; doi:10.1186/s13287-021-02255-3)
Supplement: Supplementary file 1 — Additional file 1: Table S1. Quantitative polymerase chain reaction primer sequences. [file 13287_2021_2255_MOESM1_ESM.docx]

**Additional Table 1 Quantitative polymerase chain reaction primer sequences.**

| **Gene Name** | **Primer sequences** |
| --- | --- |
| *Bcl2* | F: TGGAGAGCGTCAACAGGGAGATG  R: GGTGTGCAGATGCCGGTTCAG |
| *Bad* | F: CGGGACAGGCAGCCAATAACAG  R: AAGCTCCTCCTCCATCCCTTCATC |
| *Bax* | F: GACGCATCCACCAAGAAGCTGAG  R: GCTGCCACACGGAAGAAGACC |
| *Casp8* | F: TCTACGGAACGGATGGGAAGGAAG  R: CAGGCACAGGCACCGCTTTC |
| *Zp3* | F: CCTCCAGGCGGAAGTCCAGAC  R: TCTCAGATAGACCGTCCACAAGGC |
| *Th* | F: CTTCTGGAACGGTACTGTGGCTAC  R: AGGAGGCATGGCGGATATACTGG |
| *Amh* | F: GACACCGCAGCCAGCACATG  R: CCGCAGAGCACGAACCAAGC |
| *Fshr* | F: GGTCTCCTTGCTGGCATTCTTGG  R: CGGAATCTCTGTCACCTTGCTGTC |
| *Gapdh* | F: TGGTGAAGGTCGGTGTGAAC  R: GACTGTGCCGTTGAACTTGC |
